# Prognostic Impact of Blood Pressure Change Patterns on Patients With Aortic Dissection After Admission

**DOI:** 10.3389/fcvm.2022.832770

**Published:** 2022-06-03

**Authors:** Zhaoyu Wu, Yixuan Li, Peng Qiu, Haichun Liu, Kai Liu, Weimin Li, Ruihua Wang, Tao Chen, Xinwu Lu

**Affiliations:** ^1^Department of Vascular Surgery, School of Medicine, Shanghai Ninth People's Hospital, Shanghai Jiao Tong University, Shanghai, China; ^2^Big Data Research Lab, University of Waterloo, Waterloo, ON, Canada; ^3^Department of Economics, University of Waterloo, Waterloo, ON, Canada; ^4^Stoppingtime (Shanghai) BigData & Technology Co., Ltd., Shanghai, China; ^5^Department of Automation, Shanghai Jiao Tong University, Shanghai, China; ^6^Ningbo Artificial Intelligent Institute, Shanghai Jiao Tong University, Ningbo, China; ^7^School of Mathematical and Computational Sciences, University of Prince Edward Island, Charlottetown, PE, Canada; ^8^Faculty of Computer Science, Dalhousie University, Halifax, NS, Canada; ^9^Senior Research Fellow of Labor and Worklife Program, Harvard University, Cambridge, MA, United States

**Keywords:** aortic dissection, blood pressure, classification, functional data analysis, adverse events

## Abstract

**Objectives:**

Hypertension is a predominant risk factor for aortic dissection (AD), and blood pressure (BP) control plays a vital role in the management of AD. However, the correlation between BP change and the prognosis for AD remains unclear. This study aims to demonstrate the impact of BP change patterns on AD prognosis.

**Methods:**

This retrospective study included AD patients at two institutions (Shanghai Ninth People's Hospital Affiliated with Shanghai Jiao Tong University School of Medicine and the Vascular Department of the First Affiliated Hospital of Anhui Medical University) between 2004 and 2018. The systolic BP (SBP) change patterns of these patients were analyzed by functional data analysis (FDA). The relationship between BP change patterns and the risk of adverse events (AEs) was assessed using survival analysis.

**Results:**

A total of 458 patients with AD were eligible for analysis. The logistic regression analysis indicated that compared with that in patients with low SBP variation (SBPV), the incidence of AEs in patients with high SBPV was significantly higher (35.84 vs. 20.35%, OR 2.19, *P* < 0.001). The patients were divided into four categories (accelerating rise, accelerating drop, decelerating rise, and decelerating drop) based on their SBP patterns after FDA fitting. The results of Kaplan–Meier analysis showed that at the 15- and 20-min time points, the incidence of AEs in the decelerating-drop group was significantly lower than that in the accelerating-rise group (OR 0.19, *P* = 0.031 and OR 0.23, *P* = 0.050). However, at the 25- and 30-min time points, the difference between these four groups was not significant (OR 0.26, *P* = 0.08 and OR 0.29, *P* = 0.10).

**Conclusions:**

This study classified AD patients into four groups according to the SBP change patterns the first 30 min following admission, of which those with accelerating rises in SBP are at the highest risk of AEs, while those with decelerating drops have the best prognosis in the first 24 h after admission. Clinical practitioners may benefit from analyzing patterns of in-hospital SBP.

## Introduction

Aortic dissection (AD) is a catastrophic aortic disease with an in-hospital mortality rate of 10–18% and a death rate of approximately 20–30% before admission to the hospital (1, 2). Hypertension is a predominant risk factor for AD because the increasing shear stress resulting from high blood pressure (BP) can lead to the initial tear in the aortic intima and the subsequent progression of AD (3), and 72.1% of AD patients have a history of hypertension (4). Thus, in the clinical management of AD, BP control has played a vital role, as hypertension (systolic BP, SBP >150 mm Hg) is associated with a higher incidence of vascular complications, and hypotension (SBP ≤ 80 mm Hg) is associated with a higher incidence of malperfusion syndromes in patients with acute AD (5, 6).

The latest clinical guidelines recommend a rapid SBP reduction to the target value of 100–120 mm Hg (7–10). However, this antihypertensive therapy has not been effective, and the mortality rate has not decreased over the last 20 years (1). There are two potential reasons for this inefficiency. First, it is unclear to what extent practitioners should control BP to optimize the survival rates of AD patients (11, 12). Several scholars found that this population-based BP control strategy was not suitable for all AD patients, where certain individuals may develop severe malperfusion syndromes due to intensive BP control (5, 11). On the other hand, a recent randomized trial revealed that intensive BP control (SBP <120 mm Hg) could significantly reduce the incidence of cardiovascular complications in hypertensive patients (12). Moreover, researchers found a negative correlation between the SBP at admission and the in-hospital mortality rate for patients with Stanford type A AD (13). Second, the fixed threshold fails to take into account the dynamic nature of BP, which is critical in the development of cardiovascular events. For instance, earlier studies have suggested that the circadian rhythm of BP fluctuation has an impact on the occurrence of AD (14–17) and that BP variations have strong predictive power for adverse events (AEs) in AD (18, 19). There is, however, no clear correlation between the pattern of change in BP and the prognosis for AD. Considering the urgency of the need to control BP in AD patients after admission, we aimed to demonstrate the impact of BP change patterns on AD prognosis and provide guidelines for the management of BP in AD patients.

## Methods

This was a retrospective, multicenter study of AD. We collected the clinical information of consecutive AD patients at two institutions (Shanghai Ninth People's Hospital Affiliated with Shanghai Jiao Tong University School of Medicine and the Vascular Department of the First Affiliated Hospital of Anhui Medical University) between January 2004 and December 2018. The need for written patient consent was waived because of the observational nature of this study. This study was registered with the Chinese Clinical Trial Registry (registration number: ChiCTR1900025818). The inclusion criteria were hospital admission for AD patients diagnosed by computed tomographic angiography (CTA). The following were used as exclusion criteria: age < 18 years, pregnancy, lack of BP record, patients with traumatic, inflammatory or iatrogenic dissections, patients with a previous aortic surgery, or incomplete medical history.

All AD patients received either conservative treatment or emergency surgery after admission, and each sample included BP data from up to 14 days. The patients' BPs were measured approximately every 15– 30 min by automated noninvasive BP monitors, and the demographic information and medical history of each patient was collected from his or her medical records. In this analysis, the presence of another acute AD in the past is considered as having an AD history, and complicated AD is defined as persistent pain, uncontrolled hypertension, early aortic expansion, malperfusion, and signs of rupture (18).

To examine the relationship between the patient's history of hypertension, in-hospital BP variation, and progression of AD, we calculated the standard deviation (SD) of SBP in each patient during his or her hospitalization and stratified them into high and low SBP variation (SBPV) groups according to the average SBPV, which is the sample mean of all patients' SBPVs. A high SBPV group consists of patients whose SBPV is greater than the average, and a low SBPV group consists of patients whose SBPV is lower than the average (20). The incidence of AE was compared between the two groups through the *t* test. The first logistic regression model considers the impact of having a history of hypertension on the patient's SBPV, and the second model determines the impact of SBPV on the incidence of AE after admission. In this paper, AEs included fatal or nonfatal aortic rupture, organ or limb ischemia, and death.

In addition, our study analyzes the relationship between the fluctuation in SBP during the patients' first 30 min at the hospital and the incidence of AEs on the first day after admission. We should note before moving forward that each patient's SBP was discretely recorded at different time points, while it indeed exits at any point in time over a continuous period of time. Thus, the underlying SBP process is a function over time intervals, and it was necessary to conduct functional data analysis (FDA) to first estimate the process before analysis (21, 22). FDA is a nonparametric and continuous analysis technique proposed by Ramsay for functional data and has been shown to be an accurate estimation tool that can automatically adapt to the correct limit and recover the true underlying structure from discretely observed data in a wide variety of fields such as biomedical science, medicine, economics, finance, linguistics, psychology and sports (18, 23–28). The complete estimation process is illustrated in the Supplementary File. With the estimated underlying process, we can then determine the patients' SBP at particular time points after admission and monitor their temporary changes. We tracked the patient's condition at four different time points: 15, 20, 25, and 30 min following admission. Finally, based on the fitted curves for SBP, we computed the first and second derivatives and classified patients into mutually exclusive groups based on their signs. The first derivative of a curve indicates the slope of the SBP curve, with positive values indicating an increasing SBP and negative values indicating a decreasing SBP. The second derivative corresponds to the curvature, with a positive value representing accelerating changes in SBP and a negative value representing decelerating changes in SBP. Taking the sign of each derivative, we categorized the patients into four groups at each predetermined time point: accelerating rise, accelerating drop, decelerating rise, and decelerating drop (24). The relationship between each BP classification and the outcomes was assessed using survival curves and the logistic regression for each of the four time points. Kaplan-Meier is a non-parametric statistic that is often used to estimate the survival function from lifetime data, and it has been used extensively in a variety of disciplines including but not limited to medical, economics, and engineering (24, 29). Survival curves were constructed using Kaplan–Meier analysis and parallels with the log-rank test. R software (http://www.r-project.org) was used for statistical analyses. Continuous variables are expressed as the mean ± SD, and categorical variables are shown as percentages. A two-tailed *p* < 0.05 implies that the statistics are significantly different.

## Results

A total of 458 patients were enrolled in the current study, and Table 1 summarizes the patients' demographical and clinical characteristics. The average age of the patients in the sample was 57.1 years; the initial presentation with chest or abdominal pain occurred in 81.2% of patients; 35% of the patients were diagnosed with Stanford type A dissection, and the rest were diagnosed with type B.

**Table 1:** Physical and clinical characteristics of the included patients.

	***n* = 458**
Age, years mean (±SD)	57.1 ± 13.4
Male	363 (79.3%)
Symptom	
0-None	42 (9.2%)
1-Pain	372 (81.2%)
2-Shock	8 (1.7%)
3-Others	36 (7.9%)
ODT, days mean (±SD)	24.7 ± 111.1
Marfan syndrome	22 (4.8%)
COPD	29 (6.3%)
Hypertension	315 (68.8%)
Diabetes mellitus	28 (6.1%)
History of AD	24 (5.2%)
Cardiac diseases	85 (18.6%)
Renal insufficiency	34 (7.4%)
PAD	15 (3.3%)
Maximum aortic diameter ≥ 5.5 cm	98 (21.4%)
Type of AD	
0-Stanford type A	161 (35.2%)
1-Stanford type B	297 (64.8%)
Complicated AD	89 (19.4%)
Pericardial effusion	43 (9.4%)
Pleural effusion	105 (22.9%)

*ODT, onset to door time; COPD, chronic obstructive pulmonary disease; AD, aortic dissection; PAD, peripheral arterial disease*.

The logistic regression analysis indicated that a history of hypertension was associated with a high SBPV (OR 1.56, *P* < 0.05). The average SBPV of all patients was 13.19. According to our SBPV classification, 173 patients were classified as having high SBPV, and 285 patients were classified as having low SBPV. Compared with that in the low SBPV group, the incidence of AEs in the high SBPV group was significantly higher (35.84 vs. 20.35%, *P* < 0.001; Table 2). Moreover, logistic regression analysis further confirmed the strong association between high SBPV and AE (OR 2.19, *P* < 0.001).

**Table 2:** The incidence of AEs in the high SBPV group vs. the low SBPV group.

	**Overall**	**High SBPV**	**Low SBPV**	***P*** **value**
	**(*n* = 458)**	**(*n* = 173)**	**(*n* = 285)**	
AE (%)	120 (26.20)	62 (35.84)	58 (20.35)	< 0.001

**Figure 1:**
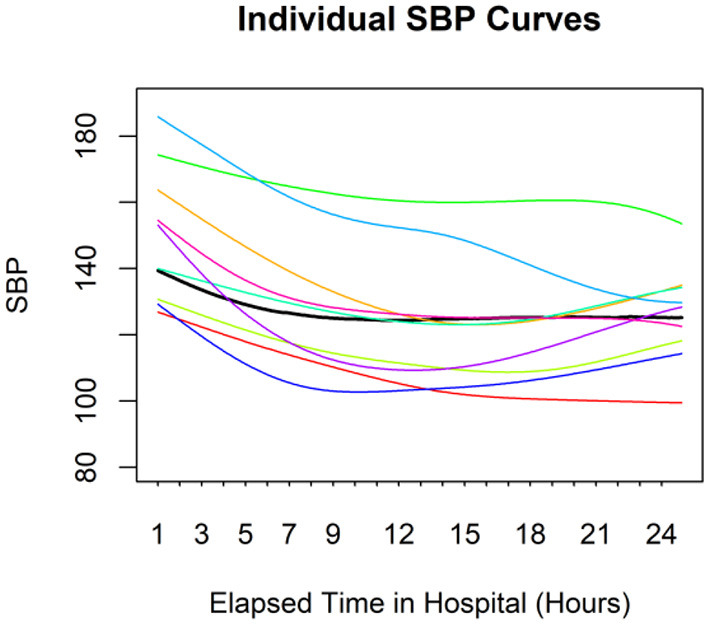
Individual SBP curves for nine random individuals. The black line represents the average curve for the entire sample. There is considerable variation among individuals.

Figure 1 shows the SBP curves for nine randomly selected individuals and the average curve for all enrolled patients during their first 24-h hospitalization. The average curve presented the BP pattern with a drop during the first 5 h, followed by a leveling off. However, the BP pattern was not consistent across all patients. The rate and degree of BP reduction after admission varied among AD patients, and some patients had progressively elevated BP after their BP dropped in the first few hours after admission.

As all patients had their BP measurements within the first 30 min after admission, this analysis included all 458 patients. Based on the SBP pattern during hospitalization after FDA fitting, we divided patients into four categories (Supplementary Figure 1). Among all patients, when the patients were classified at 15 min, 80.57% of them had SBP drops: 19.65% with decelerating drops and 60.92% with accelerating drops (Table 3). The proportion of patients in the accelerating-drop group increased, while the proportion of patients in the decelerating-drop group decreased with the extension of admission time. More Stanford type B patients were found in the Accelerating-drop and Decelerating-drop groups, and there were equal numbers of Stanford type A and type B patients in the Accelerating-rise and Decelerating-rise groups (Supplementary Table 1).

**Table 3:** Distribution of AD patients after SBP classification.

	**Accelerating**	**Accelerating**	**Decelerating**	**Decelerating**
	**rise**	**drop**	**rise**	**drop**
15 min	7.21%	60.92%	12.23%	19.65%
20 min	7.64%	62.45%	11.79%	18.12%
25 min	7.64%	65.07%	11.57%	15.72%
30 min	7.86%	65.94%	11.35%	14.85%

**Figure 2:**
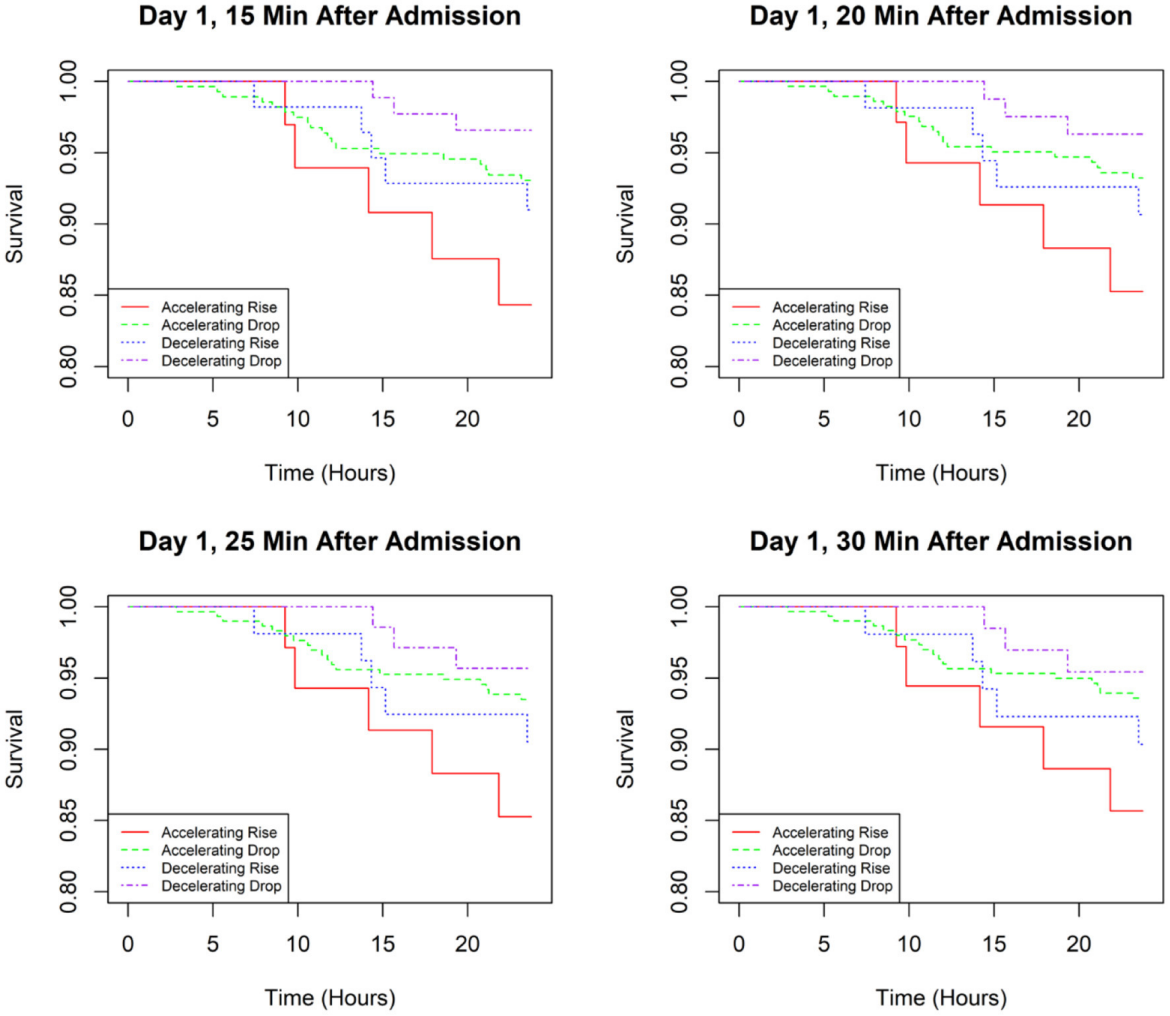
Kaplan–Meier curves for time to an adverse event in the first 24 h of admission based on SBP classifications at different time points.

Kaplan–Meier analysis was performed to determine the association between BP pattern at 15, 20, 25, and 30 min after admission and the prognosis of AD during the first 24 h of hospitalization (Figure 2). The results showed that patients with an accelerating rise at these four time points had the highest risk of AEs. In contrast, patients in the decelerating-drop group had the lowest incidence of AEs. At the 15- and 20-min time points, logistic regression analysis revealed that the incidence of AEs in the decelerating-drop group was significantly lower than that in the accelerating-rise group (Table 4). However, at the 25- and 30-min time points, the difference between these four groups was not significant. The subgroup analyses among patients with acute AD were conducted as well, and the results were consistent with that of the overall population (Supplementary Figure 2 and Supplementary Table 2).

**Table 4:** The risk of AEs compared with the accelerating-rise group.

	**15 min**	**20 min**	**25 min**	**30 min**
Accelerating rise	/	/	/	/
Accelerating drop	0.41 (0.10)	0.43 (0.11)	0.41 (0.10)	0.42 (0.10)
Decelerating rise	0.53 (0.35)	0.59 (0.43)	0.60 (0.45)	0.64 (0.51)
Decelerating drop	0.19 (0.03)	0.21 (0.04)	0.25 (0.07)	0.27 (0.09)

*The values indicate the odds ratio (p value). The results were adjusted for Marfan syndrome and cardiac diseases*.

## Discussions

### Summary of Main Results

The current study demonstrates that when AD patients are classified based on their in-hospital SBPV, patients with high SBPVs are at higher risk of AEs during hospitalization. Furthermore, when the patients were classified into four groups according to their SBP patterns during the 30-min period after admission, the results of the Kaplan–Meier analysis revealed that patients with an accelerating rise had the highest risk of AEs, while patients in the decelerating-drop group had the best prognosis during the first 24 h after admission.

### The Review in the Context of Other Literature

Hypertension poses a substantial risk for AD, as shear stress associated with high BP can cause a tear in the aortic intima (30). Earlier studies indicated that the incidence of AD varied within a day in that the highest incidence occurred between 6:00 am and 12:00 am (31). The chronobiological patterns of AD onset were consistent with the circadian variation in BP (14), and patients who lack a nocturnal BP fall may have a higher risk of AD (17). These results indicated that the circadian rhythm of BP fluctuations had impacts on the occurrence of AD. In their study, Mehta et al. demonstrated that the incidence of AEs and in-hospital mortality of acute AD patients was not different among four different time periods within 1 day (0:00 am−6:00 am, 6:00 am−12:00 pm, 12:00 pm−6:00 pm, 6:00 pm−12:00 am) (32). Since circadian changes in BP reflect short-term fluctuations in BP, subsequent researchers began examining the relationship between BP variation and the development and progression of AD. Karatas et al. demonstrated that compared with hypertensive patients without AD, AD patients had significantly higher variations in BP, although the 24-h mean BP was similar between the two groups (33). Zhang et al. analyzed BP variation before endovascular therapy in AD patients and found that patients with high BP variation had an apparently higher incidence of aorta-related mortality (28.4 vs. 9.1%) (19). In addition, the thrombosis ratio of the false lumen was significantly lower in the high BPV group at the 6-month follow-up (86.4 vs. 69.7%) (19). Qiu et al. showed the association between unit increases in BP and the incidence of in-hospital AEs in AD patients by FDA (18). In the current study, based on our multicenter data, we found that patients with a history of hypertension were more likely to have high SBPV, which was further associated with an increased incidence of AEs. These findings could be explained by the abnormal BP rhythm and poor BP control and were in line with the results of previous studies (17, 19, 33). There is a challenge, however, in estimating the average SBPV and classifying the patients into high and low SBPV groups given different target populations in clinical practice; additionally, when using only the SD of the SBP as a summary measure, there is a loss of information. Otherwise, once these high SBPV patients are identified, it is unclear what the appropriate BP control strategy should be. For these reasons, a more specific and detailed classification of BP fluctuation patterns in AD patients is of great significance for guiding clinical practice.

Presently, the antihypertensive therapy of AD is controversial in that the current clinical guidelines of BP control lack high-level clinical evidence (7–10), and clinical data have indicated no significant improvement in the efficacy of antihypertensive therapy for AD over the last two decades (1). Several studies found that the target BP level of 100–120 mm Hg was not suitable for all AD patients because rapid intensive BP lowering may lead to organ ischemia for patients with high basal BP (11, 34, 35). Therefore, individualized antihypertensive therapy for AD patients is essential. However, there is a lack of screening methods for high-risk patients that may be useful in developing a sufficient BP control strategy. In this paper, we classified AD patients into four groups based on their SBP patterns at four time points (15, 20, 25, and 30 min) after admission and conducted a predictive analysis for these patients. Patients with SBP increases, especially accelerating rises at the four time points, exhibited the highest incidence of AEs during the first 24 h after admission, suggesting that the analysis of BP patterns during the first 30 min after admission is helpful in identifying high-risk AD patients. Moreover, the risk of AEs was comparable between the decelerating-rise and accelerating-drop groups, which might result from the differences in the pathogenesis of aortic rupture and ischemia complications. Specifically, a continuous increase in SBP may lead to AD progression, while a rapid reduction in BP may result in organ hypoperfusion. It was also noteworthy that patients in the decelerating-drop group exhibited the best prognosis at 24 h after admission, which implied that steady and stable BP reduction may be applicable in clinical settings.

Based on the results of the present study, we can prescreen patients at high risk for vascular and organ complications with their SBP patterns during the first 30 min after admission. Moreover, close monitoring and appropriate SBP control strategies can be applied to these patients. For patients with persistently elevated SBP after admission, we can adjust the type and dose of antihypertensive drugs to gradually lower their SBP. In addition, resistant hypertension, defined as SBP ≧ 135/80 mm Hg despite the prescription of at least three antihypertensives, is sometimes used as an indication for surgery (36, 37). Nevertheless, the current definition of resistant hypertension does not reflect the dynamic process of BP. Based on our classifications, the pattern of BP changes that are characterized as accelerated rises or decelerating rises after medical treatment may be more consistent with the nature of resistant hypertension.

### Limitations

There are several limitations to this work. First, there is the possibility of bias due to the observational nature of this study, and the causal relationship between changes in BP and the occurrence of AEs is unclear. To establish causation between the two variables, a prospective database with randomized controlled experiments should be utilized. Second, the sample size was relatively small, primarily due to the low incidence and high prehospital mortality rate of AD. Further studies with larger sample sizes are warranted to replicate these preliminary findings. Third, most patients included in this study were Chinese; therefore, caution should be exercised in extrapolating our findings to other ethnic groups. Forth, continuous monitoring of BP is imperative for analyzing the changing pattern of BP after admission, which is not taken sporadically as laboratory tests. This may be difficult to implement in some clinical situations. Finally, the effect of the BP fluctuation pattern on the mid- and long-term prognosis of AD patients remains unclear and requires further investigation.

## Conclusions

The current study confirms that SBPV is associated with the prognosis of AD. In addition, AD patients can be classified into four groups according to the patterns of changes in SBP exhibited during the first 30 min following admission, of which those with accelerating rises in SBP are at the highest risk of AE, while those with decelerating drops have the best prognosis in the first 24 h after admission. In light of these results, it appears that clinical practitioners may benefit from analyzing patterns of in-hospital SBP at different time points. These results need to be verified in a large-sample prospective AD database.

## Data Availability Statement

The raw data supporting the conclusions of this article will be made available by the authors, without undue reservation.

## Ethics Statement

The studies involving human participants were reviewed and approved by Institutional Review Board of Shanghai Ninth People's Hospital. The Ethics Committee waived the requirement of written informed consent for participation.

## Author Contributions

WL, RW, TC, and XL led in the conception and design of the study, revised the manuscript, supervised, validated the clinical work, and results. ZW, YL, and PQ collected research data, performed the statistical analysis, and drafted the manuscript. KL and HL revised the manuscript. All authors have read and agreed to the published version of the manuscript.

## Funding

This work was supported by the National Natural Science Foundation of China (51890892 and 81971712), Clinical Research Program of Shanghai Ninth People's Hospital (JYLJ202010), Shanghai Science and Technology Innovation Action Plan (20Y11909600 and 21S31904300), Clinical Research Plan of SHDC (SHDC2020CR6016-003), Shanghai Ninth People's Hospital Nursing Fund Project (JYHL2020MS01), Shanghai Municipal Health Bureau Project (202040434), Open Research Program of National Facility for Translational Medicine (Shanghai) (No. TMSK-2021-121), and Fundamental research program funding of Shanghai Ninth People's Hospital Affiliated to Shanghai Jiao Tong university School of Medicine (JXZZ153).

## Conflict of Interest

YL was employed by Stoppingtime (Shanghai) BigData & Technology Co., Ltd., Shanghai, China. The remaining authors declare that the research was conducted in the absence of any commercial or financial relationships that could be construed as a potential conflict of interest.

## Publisher's Note

All claims expressed in this article are solely those of the authors and do not necessarily represent those of their affiliated organizations, or those of the publisher, the editors and the reviewers. Any product that may be evaluated in this article, or claim that may be made by its manufacturer, is not guaranteed or endorsed by the publisher.
