# Supplementary material for: Prognostic Impact of Blood Pressure Change Patterns on Patients With Aortic Dissection After Admission
Source: Front Cardiovasc Med. 2022 Jun 3;9:832770. doi: 10.3389/fcvm.2022.832770 (PMC9204146; doi:10.3389/fcvm.2022.832770)
Supplement: Supplementary file 1 [file Data_Sheet_1.pdf]

APPENDIX

METHODS

Estimation of the Underlying SBP Process using the Functional Data Analysis

Let $X_{i,t}$ be the value of the SBP observed at time t for patient i. Assuming the smoothness in the underlying process of SBP, denoted $W_i(t)$, we express $X_{i,t}$ as

$$X_{i,t} = W_i(t) + \epsilon_i(t),$$

where $\epsilon_i(t)$ is a random error with mean zero and finite variance. Based on the standard leave-one-out cross-validation (CV) method,[1] we select the optimal number of basis functions (H) to be 107 and the optimal smoothing parameters (γ) to be 1.29155×10^{-9}. Then, $W_i(t)$ can be approximated by

$$W_i(t) \approx \sum_{h=1}^{H} c_{i,h} \phi_{i,h}(t),$$

where the coefficients $c_{i,h}$ are obtained based on the parameters (H, γ) and $\phi_{i,h}(t)$ is the basis functions.[1]

RESULTS

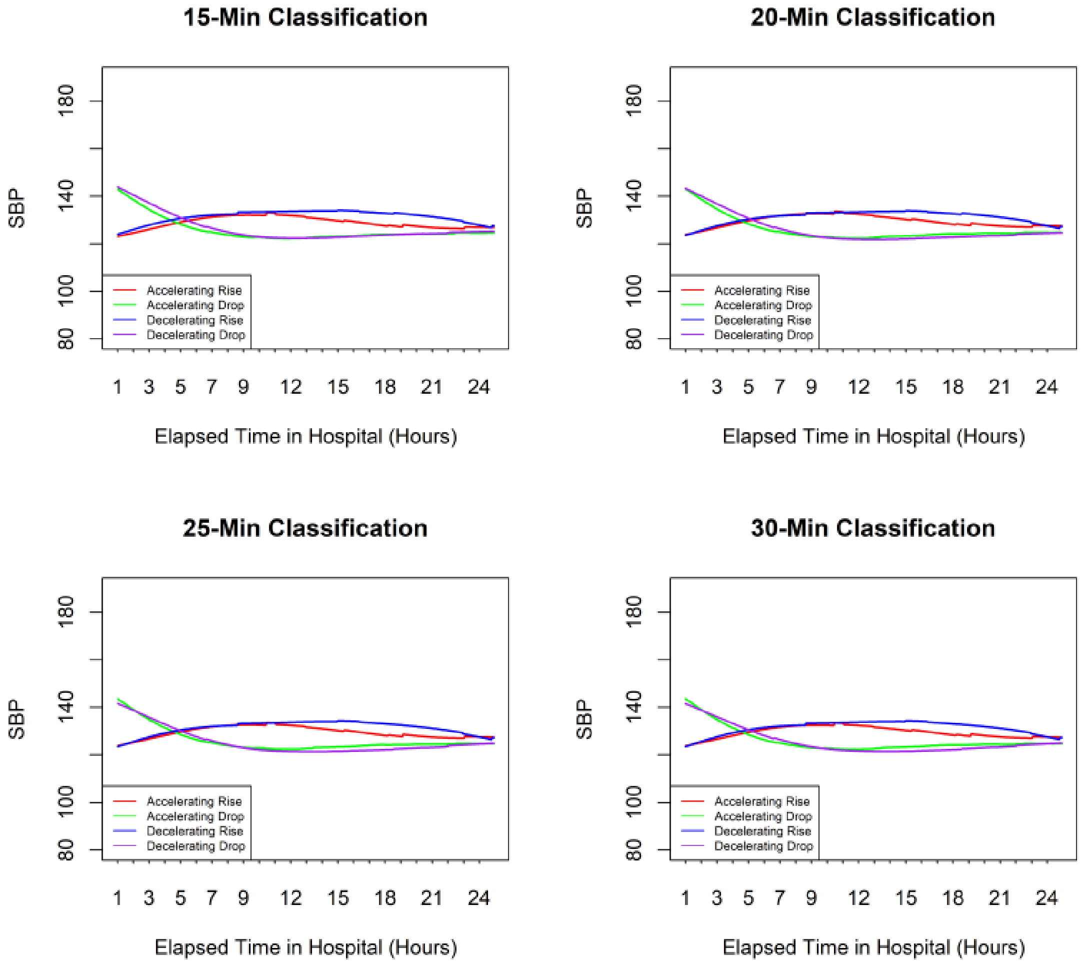

Supplementary Figure 1. The average SBP curves for individuals at 15, 20, 25, and 30 minutes.

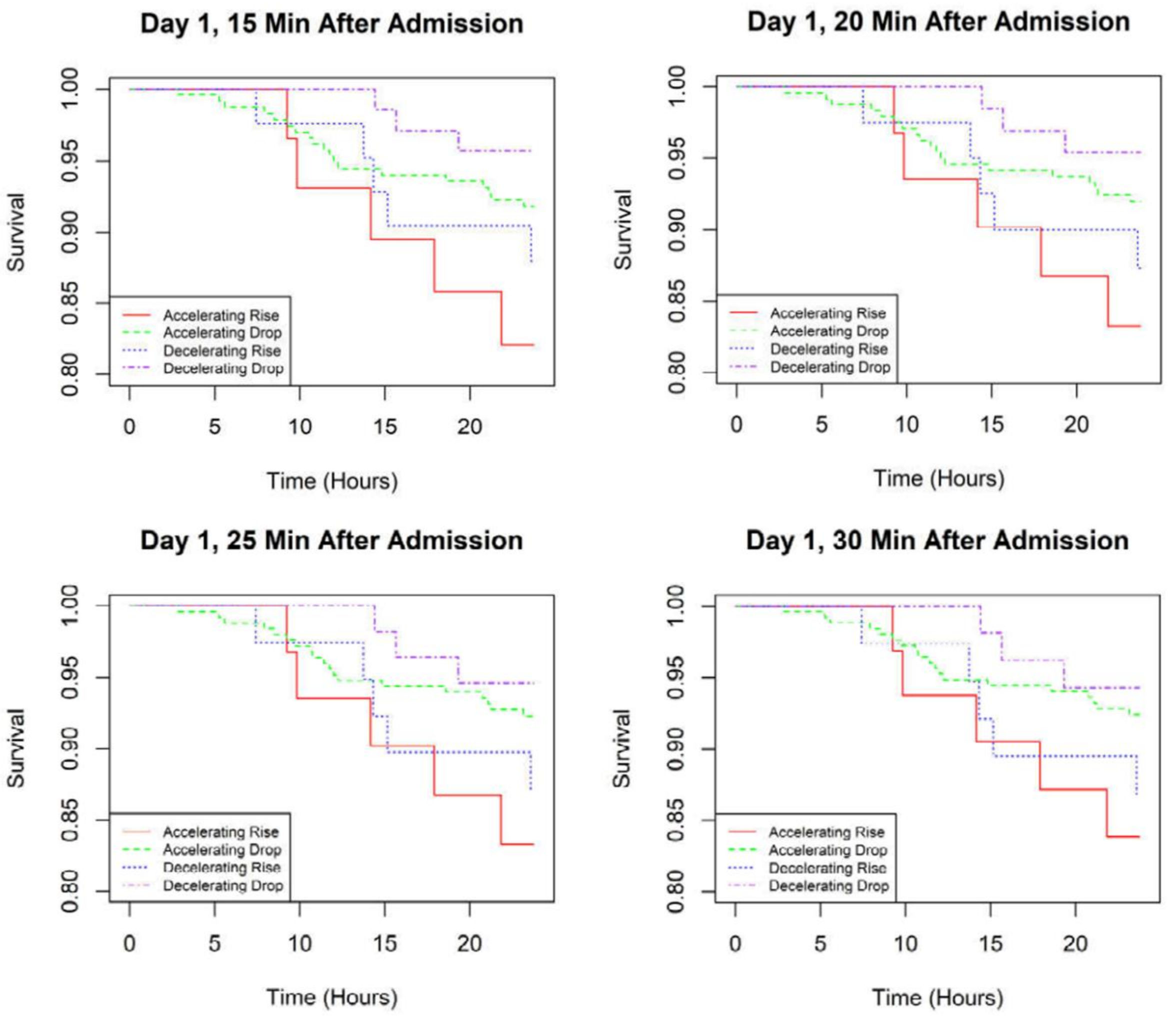

Supplementary Figure 2. The Kaplan-Meier curves for time to an adverse event in the first 24-hour after admission based on SBP classifications in acute AD patients.

Supplementary Table 1. The number of type A and type B AD patients in the four subgroups at different time points.

Time point	Type	Accelerating Rise	Accelerating Drop	Decelerating Rise	Decelerating Drop
15min	A	17	95	28	21
	B	16	184	28	69
20min	A	18	96	27	20
	B	17	190	27	63
25min	A	18	96	26	21
	B	17	202	27	51
30min	A	18	97	26	20
	B	18	205	26	48

Supplementary Table 2. The risk of AEs compared with the accelerating-rise group in acute AD patients

	15 min	20 min	25 min	30 min
Accelerating Rise	/	/	/	/
Accelerating Drop	0.42 (0.11)	0.44 (0.13)	0.42 (0.11)	0.44 (0.12)
Decelerating Rise	0.65 (0.53)	0.74 (0.66)	0.77 (0.70)	0.82 (0.77)
Decelerating Drop	0.21 (0.04)	0.24 (0.07)	0.28 (0.10)	0.31 (0.13)

The values indicate the odds ratio (p value).

REFERENCES

1. Ramsay James. *Functional Data Analysis.* 2nd edition. Springer. 37-57.
